# Supplementary material for: Scalp dose analysis for transient and permanent alopecia following conventional cranial irradiation using Image Guided Radiotherapy (IGRT): A prospective study
Source: PLoS One. 2024 Oct 10;19(10):e0311855. doi: 10.1371/journal.pone.0311855 (PMC11466407; doi:10.1371/journal.pone.0311855)
Supplement: Attachment — Submitted filename: Response to Reviewers.docx [file pone.0311855.s002.docx]

**RE:** PONE-D-23-41670

**Dear** Academic Editor PLOS ONE

I would like to thank you for sending the comments of the reviewers following the peer review of our Original Article entitled “Scalp dose analysis for transient and permanent alopecia following conventional cranial irradiation using Image Guided Radiotherapy (IGRT): A prospective study”

Please find attached the revised manuscript. We have addressed the reviewers’ comments in italic. All changes are highlighted.

***Review Comments to the Author***

***Reviewer #1***

*The manuscript is good interesting and good designed. Introduction is good written. Material and methods are good designed. Results are good written and illustrated. Discussion is good written and include the conclusion of the study.*

**Response:**	Thank you for your kind comments. Additionally, this manuscript has been revised in accordance with the formatting guidelines of PLOS ONE.

Sincerely,

Imjai Chitapanarux
