# Supplementary material for: Scalp dose analysis for transient and permanent alopecia following conventional cranial irradiation using Image Guided Radiotherapy (IGRT): A prospective study
Source: PLoS One. 2024 Oct 10;19(10):e0311855. doi: 10.1371/journal.pone.0311855 (PMC11466407; doi:10.1371/journal.pone.0311855)
Supplement: Attachment — Submitted filename: Response to Reviewers.docx [file pone.0311855.s003.docx]

August 20, 2024

**RE:** PONE-D-23-41670

**Dear** Academic Editor PLOS ONE

I would like to thank you for sending the comments of the reviewers following the peer review of our Original Article entitled “Scalp dose analysis for transient and permanent alopecia following conventional cranial irradiation using Image Guided Radiotherapy (IGRT): A prospective study”

Please find attached the revised manuscript. We have addressed the reviewers’ comments in the order they were provided, the reviewer’s comments are in italic. All changes are highlighted. According to the changes the in manuscript, we have also modified tables as follows:

Table 2 has been revised to “The dosimetric parameter of the entire scalp volume that predicted the occurrence of alopecia” using ROC curve analysis.

We have converted the previous Table 2 to Table 3, and Table 3 to Table 4.

***Review Comments to the Author***

***Reviewer 1***

*The manuscript is good interesting and good designed. Introduction is good written. Material and methods are good designed. Results are good written and illustrated. Discussion is good written and include the conclusion of the study.*

**Response:**	Thank you for your kind comments. Additionally, this manuscript has been revised in accordance with the formatting guidelines of PLOS ONE and stated the Ethics statement at the beginning of the Methods.

***Reviewer 2***

***Strengths
Study design:*** *The prospective study design is robust and the inclusion/exclusion criteria are well defined. The use of detailed dosimetric analysis for both the entire scalp and localized areas of hair loss is commendable.*

***Results:*** *Clear and concise presentation of results with appropriate descriptive statistical analysis. Differentiation between transient and permanent alopecia provides valuable insights.*

***Major review:***

*1. Definition of permanent alopecia: The manuscript defines permanent alopecia as hair loss assessed at 6 months after RT. However, permanent alopecia is typically defined as hair loss that persists for more than 9-12 months. This discrepancy should be addressed in the discussion or the results should be modified taking into account the time cutoff.*

**Response:**	Thank you for your impressive advice. The definition of permanent alopecia has been discussed. Please see the discussion session [Page 16-17, Line 253-260].

*2. I don't understand the "Sample size" paragraph, the only steady-state analyses performed are univariate so why did the authors bother to include Harris' recommendations for independent variables typically used for multivariate analyses?*

**Response:**	Thank you for your comment. Initially, we suspected that clinical alopecia may be influenced by a variety of factors. Subsequently, it is imperative to implement both univariate and multivariate analyses. Consequently, we determined the sample size in accordance with Harris' recommendations. However, after we analyzed all characteristics, only the radiation dose factor was associated with clinical alopecia. Therefore, this study did not implement any multivariable analysis.

*3. Use of ROC curve analysis: To define dose cutoffs, ROC curve analysis and AUC should be included in the study.*

**Response:**	Thank you for this important recommendation. The ROC curve analysis has been implemented, as shown in Table 2 [Page 12]. We have also made modifications to the abstract, the statistical analysis section, the result, and the discussion in accordance with your advice.

*4. English careful proofreading and possibly professional editing is recommended to refine grammar and simplify complex sentences for better readability.*

**Response:**	Thank you for your comment. We have revised English in this manuscript with professional editing.

***Minor review:***

*1. The manuscript does not clearly explain the specific advantages of IGRT over nonimage-guided radiotherapy. It should be explicitly stated and explained in the context of alopecia minimization.*

**Response:**	Thank you for your comment. This critical issue has been addressed in the discussion session [Page 17, Line 261-269]. The radiation technique utilized in this research was IGRT, as it was implemented in all curative-intent radiotherapy at our center.

*2. Median follow-up time should be entered*

**Response:**	Thank you for your suggestion. The median follow-up time has been stated in the result session [Page 10, Line 162].

*3. It would have been interesting to also include other factors that may justify influencing toxicity i.e., cigarette smoking, tumor target volume and RT techniques used (VMAT, IMRT etc)*

**Response:**	Thank you for your advice. The tumor target volume and RT techniques have been examined. Please refer to Table 1 for the data. To our regret, the cigarette consumption was not document as a patient characteristic.

***Reviewer 3***

*In my opinion, this is an interesting prospective study with appropriate method and statical analysis. It is well written on general knowkedge and previous studies about this theme. Totally, this is a good manuscript. I have one comment about statical analysis.
In discussion, authors mentions the V20 and V40 of scalp. Have you considered obtaining these parameters and comparing them with D2, Dmean, and D50?*

**Response:**	Thank you for your suggestion. V20 and V40 to the entire scalp have been further analyzed and discussed, following your advice. Please see the additional data in Tables 1 to 4, as well as the discussion [Page 15, Line 222-230] and the conclusion [Page 19, Line 285-286].

We thank you for your careful review.

Sincerely,

Imjai Chitapanarux
